# Members of the League of Mercy at Marlborough House

**Published:** 1904-06-11

**Authors:** 


### MEMBERS OF THE LEAGUE OF MERCY AT MARLBOROUGH HOUSE.

Last Wednesday afternoon a reception of the members of the League of Mercy was held at Marlborough House.

The League, incorporated by Royal Charter, promotes support for King Edward's Hospital Fund by obtaining small subscriptions. In connection with the League the Order of Mercy has been instituted by His Majesty the King (Patron of the League and Sovereign of the Order) as a reward for distinguished personal service, and as Grand President and Lady Grand President the Prince and Princess of Wales prove their practical interest in the society by thus graciously inviting the members to Marlborough House.

At 3 o'clock some hundred of the presidents and lady presidents of the League assembled in the famous saloon, where Mr. J. Harrison, M.V.O., one of the hon. secretaries, presented to their Royal Highnesses the report for the year. Colonel Knollys, the Registrar of the League, then read the following list of those to whom the Order had been awarded during the past year by the King:—

H.R.H. Princess Alexander of Teck. (Lady President, Epsom—Esher.)
H.H. Princess Victoria of Schleswig-Holstein. (Lady President, Berkshire.)
The Countess of Dartmouth. (Lady President, Lewisham.)
The Lady Harris. (Lady President, Faversham.)
The Lady Florence Pelham-Clinton (representing H.R.H. the Duchess of Albany, Lady President, Deptford—Brockley.)
Mrs. Charles Wightman. (Lady President, Hampstead.)
Col. Sir Edwin Hughes. (President, Woolwich.)
Montagu Sharpe, Esq., D.L., J.P., C.A. (President, Brentford.)
Mrs. Bentall. (Lady Vice-President, Maldon.)
Mrs. Pritchard Binnie. (Lady Vice-President, South Paddington.)
Mrs. Bishop. (Lady Vice-President, Woolwich.)
Mrs. Gerald Buxton. (Lady Vice-President, Epping.)
Mrs. Castle. (Lady Vice-President, Kingston—Surbiton.)
Miss Cayley. (Lady Vice-President, Fulham.)
Mrs. Chancellor. (Lady Vice-President, Richmond.)
Mrs. N. Cox. (Lady Vice President, Fulham.)
Mrs. Dawson. (Lady Vice-President, Woolwich.)
Mrs. Norfolk. (Lady Vice-President, Deptford—Brockley.)
Miss Swindells. (Lady Vice-President, Lambeth—Norwood.)
Mrs. Wilkinson. (Lady Vice-President, North St. Pancras.)
Sir Theodore Martin, K.C.B., K.C.V.O. (Vice-President, Guildford.)
Wenman Blake, Esq. (Vice-President, Fulham.)
Capt. T. W. Gunton. (Vice-President, North St. Pancras.)
R. G. Edwal Locke, Esq. (Vice-President, Faversham.)
Randall Mercer, Esq (Vice-President, Medway.)
H. G. Thompson, Esq., M.D. (Vice-President, Croydon.)
Frank Turner, Esq. (Vice-President, West Marylebone.)
The Marchioness Cassar de Sain. (Member St. George, Hanover Square.)
Miss Katie Browne. (Member, Fulham.)
Miss Dennistoun. (Member, Westminster.)
Mrs. Walter Farquhar. (Member, Westminster.)
Miss C. M. Gibbs. (Member, North Hackney.)
Miss Mildred Hope. (Member, Westminster.)
Mrs. Macnab. (Member, South Paddington.)
Mrs. Payne. (Member, Colchester.)
Mrs. Skinner. (Member, Epping.)
Mrs. R. Thorne Waite. (Member, North St. Pancras.)
G. W. Boorman, Esq. (Member, Medway.)
J. Smewing, Esq. (Member, North St. Pancras.)

The Prince of Wales cordially thanked the presidents and other workers of the League for the services they had rendered through it to the London hospitals in the following words:—

"The Princess and I are very glad to have the pleasure of meeting you here in our house this afternoon, and we wish to take this opportunity of most warmly thanking you and all those who have worked with you on behalf of the League during the past year.

"From the report which we have just heard read I think we can congratulate ourselves that steady progress is being made, and I trust that the work of the League is gradually becoming better known and appreciated by the public.

"As President of King Edward's Fund I wish to express my gratitude for the very handsome sum of £10,000 received from the League at the beginning of the year."

Then the Royal party and the ladies and gentlemen who were at the meeting adjourned to the garden, where over 1,000 of the vice-presidents and members of the League were assembled. The band of the Royal Marine Light Infantry (Chatham Division) was in attendance and played the National Anthem as their Royal Highnesses, accompanied by the Countess of Airlie and Commander Sir Charles Cust, entered the grounds from the house. Their Royal Highnesses made a promenade of the garden, receiving the salutations of the assembled guests, and then took up their position at the steps leading to the house. An investiture of the "Order" then took place, Lord Wolverton presenting some 37 ladies and gentlemen to the Prince, who decorated them with the Order and shook hands with them.

The Princess also shook hands with those whom she knew personally. Her Royal Highness wore a becoming princess gown of black velvet-brocade, a black feather boa, and a toque of black jet. Prince Edward and Prince Albert and little Princess Mary of Wales accompanied the Princess. The young Princes were dressed in sailor suits and cloth caps, while the golden-haired little Princess wore a pretty white silk dress and white hat. Later in the afternoon the two youngest of the Royal children were to be seen at a front window of the palace.

Amongst the Presidents and Lady Presidents of the League of Mercy and other distinguished persons invited were:—The Duke and Duchess of Norfolk, His Grace the Duke of Argyll, K.T., G.C.V.O.; the Marquis Camden, the Earl and Countess of Dartmouth, the Earl of Clarendon, G.C.B.; the Earl of Mansfield, the Earl Cadogan, K.G, and Countess Cadogan; the Earl and Countess of Verulam, the Earl of Kilmorey, K.P., A.D.C.; the Lord and Lady Boston, the Viscount and Viscountess Gage, the Lord Medway, the Lord Hugh Cecil, M.P.; the Lord and Lady Battersea, the Lord and Lady Decies, the Lord Harris, G.C.S.I, G.C.I.E., and Lady Harris; the Lord Northbourne, the Lord Farquhar, G.C.V.O, and Lady Farquhar; H.R.H. the Duchess of Albany, H.R.H. Princess Alexander of Teck, H.H. Princess Victoria of Schleswig-Holstein, the Duchess of Somerset, the Countess Grosvenor, the Lady Florence Pelham-Clinton, Dora Countess of Chesterfield, Georgina Countess of Dudley, Viscountess Torrington, the Lady Amherst of Hackney, the Lady Edith King-Noel, the Dowager Lady Lamington, the Lady Llangattock, the Lady Petre, the Lady Pirbright, the Lady Edith Villiers, the Lady Leucha Warner, the Lady Wantage, the Hon. Mrs. Halford, the Hon. Mrs. Leveson-Gower, the Hon. Mrs. Freeman Thomas, Lady Burdett.

The Council, the members of the Visiting Committee, of the Executive Committee and of the Distribution Committees of King Edward's Hospital Fund for London were also invited:—The Duke and Duchess of Fife, the Duke and Duchess of Bedford, the Bishop of London, Viscount and Viscountess Duncannon, Lord and Lady Cheylesmore, Lord and Lady Iveagh, Lord Lister, O.M.,

Lord and Lady Oranmore and Browne, Lord and Lady Rothschild, Lord and Lady Strathcona and Mount Royal, the Postmaster-General, the Chairman of the London County Council and Mrs. Benn, the Governor of the Bank of England and Mrs. Morley, the President of the Royal College of Surgeons and Mrs. Tweedy, the Right Hon. C. Stuart Wortley, K.C., M.P., Sir John Aird, Bart., M.P., and Lady Aird, Sir W. S. and Lady Church, the Right Hon. Sir Savile Crossley, Bart., M P., and Lady Crossley.

Tea was served under a verandah-fashioned awning, and beautiful bouquets of purple, yellow, and white iris adorned the tables. A tent with small tables was reserved for the Royal hosts and their specially-favoured guests.

After tea the Prince and Princess again moved about among their guests, speaking to many of them, and when the band played "God Save the King" the visitors rose to leave, feeling charmed with the gracious reception and stimulated by it to help still further all the sick and needy who cannot help themselves.

